# Vaccination Coverage and Factors Associated With Incomplete Vaccination Schedules in Children Under 5 in a Peripheral Area of the Federal District of Brazil

**DOI:** 10.1111/phn.70066

**Published:** 2026-01-29

**Authors:** Ivea Rayane Mendes Nicacio Viana, Henry Maia Peixoto

**Affiliations:** ^1^ Centre for Tropical Medicine University of Brasília (UnB), University Campus Darcy Ribeiro Brasília DF Brazil; ^2^ Institute for Health Assessment and Translation for Chronic and Neglected Diseases of High Relevance ‐ IATS‐CARE Belo Horizonte Minas Gerais Brazil

**Keywords:** health policy, primary health care, vaccination coverage, vaccines

## Abstract

**Objective:**

To estimate vaccination coverage (VC) and analyze the factors associated with the incomplete vaccination schedule (IVS) in children under 5 years of age in two Basic Health Units in the Federal District of Brazil.

**Design:**

A cross‐sectional study.

**Sample:**

The study included 162 children in two Basic Health Units; 54.94% were male, and all were under 5 years of age.

**Measurements:**

Guardians were interviewed using a structured questionnaire, and their vaccination booklets were photographed. VC was estimated and prevalence ratios (PR) were calculated using Poisson regression analysis.

**Results:**

Twenty percent of the children had an IVS. None of the vaccines evaluated reached the VC considered correct in terms of age and interval between doses. Factors associated with IVS were age under 2 years (adjusted PR: 2.55; 95% CI: 1.53–4.24), difficulties arising from the COVID‐19 pandemic (adjusted PR: 2.71; 95% CI: 1.51–4.86) and a negative response when asked whether all vaccines were administered in the same location (adjusted PR: 1.97; 95% CI: 1.13–3.44).

**Conclusions:**

A high proportion of children presented IVS, associated with factors such as age, lack of continuity of vaccination in the same location and difficulties caused by the COVID‐19 pandemic.

## 1 Background

Immunization is the process by which a person develops resistance to an infectious disease. This protection is generally achieved through the administration of vaccines, which stimulate the immune system to produce antibodies as a defense against infections. Vaccines are widely recognized as one of the greatest revolutions in the history of medicine, due to their significant impact on both individual and collective health (Ministério da Saúde 2014; Possas et al. 2020; Barbieri et al. 2020).

Currently, several countries have adopted numerous vaccines in their vaccination schedules that work to control various infectious diseases. In Brazil, the Public Health System (Sistema Único de Saúde—SUS) offers around 20 vaccines according to the National Vaccination Schedule, benefiting children, adolescents, adults, the elderly, and pregnant women (Secretaria de Saúde do Distrito Federal 2021)

When vaccines achieve widespread application and high vaccination coverage, they inhibit the spread of infectious diseases, benefiting all individuals' health, even those who have not received the vaccines, as they gain indirect protection through herd immunity (Domingues and da Silva Teixeira 2013; Ashby and Best 2021).

The success of immunization in the face of the eradication of smallpox in 1980 in the world and the elimination of polio in 1994 and measles in 2016 in Brazil, historically contributed to the population's adherence to government vaccination programs (Possas et al. 2020; Domingues et al. 2012). However, Brazil has faced difficulties in maintaining high vaccination coverage (VC), facing challenges such as virus recirculation and an increase in the growth of anti‐vaccine movements in recent years (Cruz 2017; França et al. 2025).

Influenced by low coverage, Brazil recorded the return of measles, with outbreaks occurring just over a year after receiving the elimination certificate from the Pan American Health Organization (PAHO) in 2016, leading the country to lose this certificate in 2019 (Possas et al. 2020). Since then, no region has recorded VCs greater than 95% for the first dose of measles vaccination. In 2020 and 2021, the highest and lowest VCs were obtained, respectively, in the South (86.2% and 84.2%) and North (66.8% and 68%) regions (Sato et al. 2023).

Furthermore, Brazil also recorded outbreaks of yellow fever in 2017, when 25% of the population at risk of contracting yellow fever, mostly children, were not vaccinated (Organización Panamericana de la Salud—OPAS 2020). These events demonstrated the importance of maintaining high VCs to avoid the reintroduction of vaccine‐preventable diseases in the country.

In addition to these historical and regional challenges, recent studies have highlighted persistent deficiencies in childhood vaccination coverage in Brazil. The National Vaccination Coverage Survey (2020) revealed that the proportion of children who received complete vaccination schedules remained below 75% in all state capitals and the Federal District (de Cantuária Tauil et al. 2024). Furthermore, Barata et al. emphasized that vaccine hesitancy is a multifaceted phenomenon, present in 52.3% of caregivers of children in Brazil, occurring more frequently among populations with lower educational attainment and greater socioeconomic vulnerability—although it affects society as a whole, regardless of socioeconomic or educational status (Barata et al. 2024).

Immunization is one of the most cost‐effective measures in the prevention of infectious diseases, directly reflecting on morbidity and mortality indicators and reducing disease‐related costs (Possas et al. 2020; Domingues and da Silva Teixeira 2013).

In Brazil, immunization services are guided by the Ministry of Health, through the National Immunization Program (PNI), with states and municipalities being responsible for their effective structuring and organization (Brasil. Ministério da Saúde 2017). It is noteworthy that the operationalization of immunization actions in the SUS occurs in vaccination rooms, located mainly in the context of Primary Health Care (PHC), a level of care especially focused on health promotion and disease prevention (Brasil. Ministério da Saúde 2017; das Graças Siqueira et al. 2017).

The vaccination room represents the final instance of the Vaccine Cold Chain (VCC) in Brazil. This chain comprises the entire logistical process for the conservation of immunobiologicals, from the producing laboratory to the end user, including the stages of receipt, storage, distribution, and transportation, with the vaccine room being responsible for carrying out the vaccination procedures (Brasil. Ministério da Saúde 2017).

It is understood that the achievement of high vaccination coverage results from the implemented actions, the technical capacity of the teams, the management and the social and health policies of each region, therefore, a continuous and regionalized assessment is essential (Barbieri et al. 2020), which, in addition to evaluating VC, considers local factors that may be associated with incomplete vaccination schedules (IVSs).

Thus, the present study aimed to evaluate vaccination coverage and factors associated with the IVS among children under 5 years of age through a household survey in two Basic Health Units (BHU) in the Federal District (DF), Brazil.

## 2 Methods

### 2.1 Study Design and Population

A cross‐sectional study was conducted with a descriptive component, in order to estimate VCs, and an analytical component that evaluated the factors associated with IVS. The research was carried out in two BHU, the first consisting of six Family Health Strategy (FHS) teams and a fixed and shared vaccination room, and the second by an FHS team without a fixed vaccination room, but with support from the vaccination every 15 days. Both units are located in the administrative region (AR) of Riacho Fundo, one of the 33 AR of the Federal District (FD), the capital of Brazil. Riacho Fundo is located in the outskirts of the FD, about 20 km from the central district of Brasília. It is important to highlight SUS provides universal and free health care throughout the Brazilian territory, including the operationalization of PHC through the FHS Strategy, which also covers vaccination services.

The study included children who were duly registered in the database of the e‐SUS Primary Care System (e‐SUS AB) of the BHU and who, at the time of the search, which took place between the months of February and March 2022, were aged between 6 months and less than 5 years and lived in the territory covered by these teams. Participants who changed address were excluded.

### 2.2 Sample

Proportional stratified sampling was carried out by FHS, with an estimated sample size of 336 children. The sample calculation considered a finite population of 1038 resident children, information obtained from the BHU records. It was considered 20% losses, 50% prevalence, and acceptable margin of error of 5%.

### 2.3 Data Collection and Measurements

To collect data, the study carried out a household survey. Parents/guardians were interviewed using a questionnaire containing questions about socioeconomic, demographic, and health service‐related characteristics. First, we captured caregiver characteristics: sex, race/color, education level, marital status, relationship to the child, family income class, number of children in the household, labor market status of the family head, housing conditions, and receipt of government transfer benefits. Next, we recorded child characteristics: sex, age group, and race/color. Finally, we included health service‐related variables—vaccination site consistency (all doses at the same unit; doses at different units) and reported COVID‐19 vaccination difficulties. ​The corresponding categories for each of the listed variables can be found in the tables presented throughout the text.

Additionally, the Brazilian version of Primary Care Assessment Tool (PCATool) for children was used (Brasil, Ministério da Saúde 2020). The PCATool measures the presence and extent of the four essential attributes and the three PHC‐derived attributes. The following attributes were evaluated: affiliation, which measures the user's bond with the healthcare team; first contact access, formed by the accessibility component (structure) and the utilization component (process), which evaluates PHC as a gateway; longitudinality, which presupposes the existence of a regular source of attention and its use over time; and comprehensiveness, which implies that primary care units must make arrangements so that the patient receives all types of health care services within their needs.

Data collection took place from September 2022 to January 2023 through an electronic questionnaire made available by the REDCap Brazil platform (Research Electronic Date Capture). Two Community Health Agents and a nurse/researcher were properly trained to apply the questionnaire and photograph the vaccination booklet.

To analyze the vaccination of children in this study, the vaccination schedule established by the PNI 2020 was considered (Box S1). The outcome variable was IVS. IVS was considered when the child did not receive any of the doses listed in Box S1. In the delayed dose assessment, the vaccine was considered to be administered 30 days or later in relation to the age recommended by the official PNI schedule. The assessment of the completeness of the vaccination schedule and vaccination delay was carried out independently by two experienced nurses, with disagreements being resolved by consensus.

### 2.4 Statistical Analysis

Relative frequencies were estimated for the categorical variables. For quantitative variables, medians and interquartile ranges (IQR) were calculated, expressed by the first and third quartiles, since the variables analyzed did not present a normal distribution. The Kolmogorov–Smirnov test was used to verify data normality. To compare quantitative variables, given that no normality was found, the Mann–Whitney *U* test was used. For qualitative variables, the chi‐square test or Fischer's Exact Test were used. The significance level adopted was 5%.

VCs were estimated and reported as point estimates with 95% confidence intervals. VCs were assessed taking into account the valid dose—the dose administered from the stated minimum age and, in the case of multiple doses, with an interval equal to or greater than the recommended minimum—and the correct dose, calculated considering the correct age of vaccine administration according to the official PNI schedule and the correct dose interval.

To analyze the PCATool attributes, a standardized score from 0 to 10 was assigned for score analysis. The data were categorized dichotomously with cutoff points of < 6.6 for a low attribute score and > 6.6 for a high attribute score (Brasil, Ministério da Saúde 2020).

The study used Prevalence Ratios (PR) and their confidence intervals (IC 95%) to estimate the associations between the independent variables and the IVS outcome. A Poisson regression analysis with robust variance adjustment was used to model the independent variables that presented a *p* value ≤ 0.20 in the bivariate analysis. The stepwise method with backward elimination of variables was used to select variables with *p* < 0.10 for the final model.

Statistical analyzes and random selection of samples by stratum were carried out using the Statistical Packages for the Social Sciences (SPSS) version 22.0 program. The sample calculation was carried out using the Epiinfo Software version 7.2 (CDC), through the StatCal—Sample Size and Power module.

## 3 Results

Visits were carried out to 336 homes, of which, after three attempts, 78 children were not found, 88 changed address, 6 guardians refused to participate, and 2 children had died. As a result, 162 guardians were interviewed.

When analyzing the characteristics of the interviewees (Table 1), it was found that 85.19% (138/162) were female, with 59.88% (97/162) declaring that they were the mother of the child. Regarding education, 56.79% (92/162) had secondary education (High School), while 75.31% (122/162) reported a monthly family income of up to 2.9 thousand reais (Classes D/E). Regarding the number of children, 66.67% (108/162) had between 1 and 2 children. Regarding the situation of the head of the family in the labor market, it was observed that 43.83% (71/162) of the interviewees occupied the position of salaried employees with a work permit.

**TABLE 1:** Characteristics of the interviewees, Brasília, Brazil, 2023.

Characteristics of the interviewee	Total: *f* (%)
Sex:	
Male	24/162 (14.81)
Female	138/162 (85.19)
Race or color:	
Yellow	7/162 (4.32)
White	33/162 (20.37)
Indigenous	2/162 (1.23)
Mixed race (*pardo*)	91/162 (56.17)
Black	29/162 (17.90)
Marital status of the person responsible:	
With a partner	121/162 (74.69)
Without a partner	41/162 (25.31)
Number of children:	
1 to 2	108/162 (66.67)
3 or more	54/162 (33.33)
Relationship between the child and the interviewee:	
Mohter	97/162 (59.88)
Father	43/162 (26.54)
Other	22/162 (13.58)
Family Income:	
Class C^a^	40/162 (24.69)
Class D/E^b^	122/162 (75.31)
Situation of the head of household in the labor market:	
Employed with a signed employment booklet	71/162 (43.83)
Self‐employed	45/162 (27.78)
Unemployed	32/162 (19.75)
Retired	1/162 (0.62)
Other	13/162 (8.02)
Education level of the head of household:	
Elementary and Middle School	27/162 (16.67)
High School	92/162 (56.79)
Higher Education	43/162 (26.54)
Housing conditions:	
Rented	53/162 (32.72)
Financed	7/162 (4.32)
Owned	95/162 (58.64)
Other	7/162 (4.32)
Characteristics of the children	Total: *f* (%)
Sex:	
Male	89/162 (54.94)
Female	73/162 (45.06)
Age:	
6 months to less than 2 years old	36/162 (22.22)
2 to less than 5 years old	126/162 (77.78)
Race or color:	
Yellow	2/162 (1.23)
White	73/162 (45.06)
Mixed race (pardo)	72/162 (44.44)
Black	15/162 (9.26)

^a^ Class C: monthly household income between R$2900 and R$7100.

^b^ Class D/E: monthly household income up to R$2900.

Regarding the characteristics of the children, 54.94% (89/162) were male. The evaluation of the vaccination schedules revealed that 19.75% (32/162) had IVS and 82.09% (133/162) had one or more records of vaccinations carried out outside the recommended age.

When those responsible were asked whether any vaccine had been administered late, only 53.09% (86/162) said yes. Among these, the main reasons were: sick child, representing 54.65% (47/86) of cases; forgetfulness, corresponding to 22.09% (19/86); the COVID‐19 pandemic, with 17.44% (15/86); difficulty in access, including opening hours and closed vaccination room, with 12.79% (11/86); lack of vaccines, with 6.98% (6/86); and fear of side effects, with 3.49% (3/86) of cases.

Regarding the distribution of vaccines (Figure S1), the vaccines with the highest proportion of doses administered at the correct age were pentavalent with 89.0% (144/162), oral human rotavirus vaccine 94.0% (153/162), inactivated polio vaccine 94.0% (152/162), and 10‐valent pneumococcal conjugate vaccine with 92.0% (149/162), applied at 2 months. It was also observed that multi‐dose vaccines had a higher proportion of doses administered at the correct age for the first doses, with a notable decrease and delay in subsequent doses.

Table 2 shows the vaccination coverage confirmed by the vaccination booklet for the valid and correct doses. The best coverage was identified in the 10‐valent pneumococcal conjugate vaccine, with a coverage of 99.4% at the valid dose, and in the oral human rotavirus vaccine, with a coverage of 77.2% at the correct dose. The coverage of other vaccines varied for valid doses between 93.0% and 98.0% and for correct doses between 53.0% and 75.0%. For correct dose, vaccine administered at the age and intervals established in the vaccination schedule, no vaccine reached the target established by the Ministry of Health, despite valid doses having achieved coverage of more than 90% in all vaccines.

**TABLE 2:** Vaccination coverage confirmed by the vaccination booklet, considering valid and correct doses, in children aged 6 months to less than 5 years old assisted by family health teams in Riacho Fundo II, Brasília, Brazil, 2023.

Vaccine	Children with valid doses	(%)/95% CI for valid dose	Children with correct dose	(%)/95% CI for correct dose	Target for correct dose (MS)
Pentavalent	160/162	98.77% (95.61–99.85)	76/162	46.91% (39.04–54.90)	95%
ORV	156/162	96.32% (92.11–98.63)	125/162	77.16% (69.92–83.38)	90%
IPV	159/162	98.15% (94.68–99.62)	86/162	53.09% (45.10–60.96)	95%
PCV10	161/162	99.38% (96.61–99.98)	122/162	75.31% (67.93–81.74)	95%
MenC	156/162	96.32% (92.11–98.63)	110/162	67.9% (60.12–75.01)	95%
Yellow fever vaccine	157/160	98.13% (94.62–99.61)	113/160	70.63% (62.92–77.55)	95%
MMR	153/157	97.45% (93.61–99.30)	102/157	64.97% (56.96–72.40)	95%
DTP	141/149	94.63% (89.70–97.65)	71/149	47.65% (39.41–55.98)	95%
OPV	144/149	96.64% (92.34–98.90)	81/149	54.36% (46.01–62.54)	95%
Hepatitis A vaccine	140/149	93.96% (88.84–97.20)	80/149	53.69% (45.35–61.89)	95%
Varicella vaccine	139/149	93.29% (88.80–96.73)	83/149	55.7% (47.35–63.83)	95%

*Note*: Pentavalent: diphtheria, tetanus, pertussis, haemophilus influenzae B and hepatitis B vaccine.

Abbreviations: DTP, diphtheria, tetanus, and pertussis vaccine; IPV, inactivated polio vaccine; MenC, Meningococcal C conjugate vaccine; MMR, measles, mumps, and rubella vaccine; OPV, Oral polio vaccine; ORV, oral human rotavirus vaccine; PCV10, 10‐valent pneumococcal conjugate vaccine.

In relation to the PHC attributes, the affiliation attribute achieved a moderate proportion of high scores (62.3%), with a median of 10 (IQR: 3–10), while the attribute first contact access—accessibility, a low score was identified in 90.7% of those assessed with a score of 4 (IQR: 3–6). On the other hand, in the attribute first contact—use, the high score reached a proportion of 78.4% and a median of 9 (IQR: 7–10). None of the attributes showed a statistically significant difference when comparing the measurements between children who presented complete and IVS (Table 3).

**TABLE 3:** Median, quartiles, and percentiles of primary care attributes, affiliation, first contact access—Accessibility and utilization, completeness, and longitudinality for the outcome incomplete vaccination schedule among children aged 6 months to less than 5 years old assisted by the FHS in Riacho Fundo, Brasília, Brazil, 2023.

Primary care attributes	Total (%)	Complete booklet	Incomplete booklet	*p*
Affiliation				
Median (IQR)	10 (3–10)	10 (3–10)	10 (3–10)	0.663
High	101/162 (62.34)	80/129 (62.02)	21/33 (63.64)	0.864
Low	61/162 (37.65)	49/129 (37.98)	12/33 (36.64)	
First contact—Accessibility				
Median (IQR)	4 (3–6)	4 (3–6)	4 (3–5)	0.261
High	15/162 (9.26)	14/129 (10.85)	1/33(3.03)	0.167
Low	147/162 (90.74)	115/129 (89.15)	32/33 (96.97)	
First contact—Utilization				
Median (IQR)	9 (7–10)	9 (7–10)	9 (7–10)	0.919
High	127/162 (78.40)	101/129 (78.29)	26/33 (78.79)	0.951
Low	35/162 (21.60)	28/129 (21.71)	7/33 (21.21)	
Completeness				
Median (IQR)	6 (4–8)	6 (4–8)	7 (6–8)	0.388
High	70/162 (43.21)	54/129 (41.86)	16/33 (44.48)	0.278
Low	72/162 (44.44)	61/129 (47.29)	11/33 (33.33)	
Longitudinality				
Median (IQR)	6 (4.75–8)	6 (4–8)	7 (5–8)	0.839
High	75/162 (46.30)	58/129 (44.96)	17/33 (51.52)	0.5
Low	87/162 (53.70)	71/129 (55.04)	16/33 (48.48)	

Table 4 presents the estimated Prevalence Ratios (PR) for the IVS outcome. It is possible to observe that a statistically significant association was not found when analyzing the following variables: sex of the child, race/color of the child, marital status of those responsible, education of those responsible, number of children, family income, receipt of benefits, presence of a room of vaccines at BHU and PHC attributes.

**TABLE 4:** Crude and adjusted prevalence ratio estimates for the outcome incomplete vaccination schedule among children aged 6 months to less than 5 years old assisted by family health strategy teams in Riacho Fundo, Brasília 2023.

Characteristics	Prevalence incomplete vaccine schedule *f* (%)	Crude PR (CI 95%)	*p* value	Adjusted PR	*p* value
Sex:					
Male	23/89 (25.84)	1.88 (0.96–3.71)	0.094	—	—
Female	10/73 (13.70)				
Race/color:					
Non‐white	21/89 (23.60)	1.44 (0.76–2.71)	0.267	—	—
White	12/73 (16.44)				
Age:					
1 (6 month to less than 2 years old)	12/23 (33.33)	2.00 (1.09–3.66)	0.025	2.55 (1.53–4.24)	0.000
2 (2 to less than 5 years old)	21/126 (16.67)				
Marital status:					
With a partner	26/121 (21.49)	1.26 (0.59–2.68)	0.551	—	—
Without a partner	7/41 (17.07)				
All vaccinations were carried out in the same place:					
No (different locations)	18/56 (32.14)	2.71 (1.24–4.16)	0.08	1.97 (1.13–3.44)	0.017
Yes (same place)	15/106 (14.15)				
Education:					
High School	20/92 (21.74)				
Higher Education	10/43 (23.26)	0.93 (0.48–1.82)	0.843	—	—
Number of children:					
3 or more	15/54 (27.78)				
1 a 2	18/108 (16.67)	1.67 (0.91–3.04)	0.096	—	—
Family Income:					
Receives up to 2.9 thousand	26/122 (21.31)				
Receives between 2.9 thousand and 7.1 thousand	7/40 (17.50)	1.22 (0.57–2.59)	0.609		
COVID‐19 pandemic has made vaccination difficult:					
Yes	20/52 (38.46)	3.25 (1.76–6.02)	0.000	2.71 (1.51–4.86)	0.001
No	13/110 (11.82)				
You receive an income transfer benefit:					
Yes	20/77 (25.97)	1.70 (0.91–3.18)	0.098		
No	13/85 (15.29)				
Have any vaccinations been administered late?					
Yes	31/133 (23.31)	3.38 (0.86–13.33)	0.082	3.46 (0.90–13.55)	0.071
No	2/29 (6.90)				
Vaccination room in the Basic Health Unit					
No	6/34 (17.65)	0.84 (0.38–1.86)	0.662	—	
Yes	27/128 (21.09)				
Affiliation					
Low score	21/101 (20.79)	1.06 (0.56–1.99)	0.864		
High score	12/61 (19.67)			—	—
Accessibility					
Low score	7/35 (20.00)				
High score	26/127 (20.47)	0.98 (0.46–2.06)	0.951	—	—
Utilization				—	—
Low score	32/147 (21.77)	3.26 (0.48–22.23)	0.227		
High score	1/15 (6.67)				
Longitudinality				—	—
Low score	16/87 (18.39)				
High score	17/75 (22.67)	0.81 (0.44–1.49)	0.501		
Completeness				—	—
Low score	11/72 (15.28)				
High score	16/70 (22.86)	0.67 (0.33–1.34)	0.225		

The age group from 6 months to less than 2 years showed a statistically significant association with IVS compared to children from 2 years to less than 5 years, with an adjusted PR of 2.55 (IC 95%: 1.53–4.24; *p* < 0.001) (Table 4). This means that in this study, children aged between 6 months and less than 2 years had a prevalence of IVS 2.55 times higher than children aged 2 to less than 5 years.

In the variable “all vaccines were carried out in the same location,” the negative answer to the question, compared to the affirmative answer, presented an adjusted PR of 1.97 (IC 95%: 1.13–3.44; *p* = 0.017) (Table 4). This indicates that children who do not always vaccinate in the same place have a prevalence of IVS 1.97 times higher than those who always vaccinate in the same place.

When analyzing the question “has the COVID‐19 pandemic made it difficult to vaccinate children,” it was found that the answer “Yes” presented a prevalence of 38.46%, while the answer “No” presented a prevalence of 11.82%. This resulted in an adjusted PR of 2.71 (95% IC: 1.51–4.86) (Table 4). Therefore, the prevalence of IVS among those who reported difficulties in vaccinating their children during the COVID‐19 pandemic was 2.71 times higher compared to those who did not report difficulties.

## 4 Discussion

Regarding the status of the vaccination schedule, this study found that 80.25% of the children assessed had a complete vaccination schedule, that is, all vaccines recommended in the national schedule were administered, regardless of the intervals between doses. However, it is quite worrying that the study identified that 19.75% of children had IVS and 82.09% had one or more vaccines administered outside the recommended age, which could lead to possible individual and collective consequences, affecting the control of vaccine‐preventable diseases in the assessed territory (Cruz 2017).

For valid doses, those applied from the minimum age indicated and if there are multiple doses, if there was an interval equal to or greater than the minimum recommended (Moraes 2007), all vaccines achieved coverage greater than 90%. However, for the correct dose, the one calculated taking into account the correct age and the correct interval between doses (Moraes 2007), the results showed low coverage, which raises concerns about the control of vaccine‐preventable diseases, considering that the correct dose of a vaccine represents the coverage with the greatest potential to be effective (Moraes 2007).

When evaluating the factors associated with IVS, the study did not identify a statistically significant association when analyzing variables historically associated with social vulnerability, such as race/color, education and family income, possibly due to the population having very homogeneous characteristics in social terms and being mostly low income. Studies carried out in the Brazilian cities of Rondonópolis (de Lima Lemos et al. 2022), in the State of Mato Grosso, and São Luís (Yokokura et al. 2013), in the State of Maranhão, identified similar results.

Although in the study carried out in São Luís (Yokokura et al. 2013), low maternal education was identified as a factor associated with IVS, with an adjusted PR of 1.67 (95% IC: 1.05–2.64), considering mothers with up to 4 years of schooling as exposure, it is noteworthy that the research found no association between race/color, family income and paternal education. The study carried out in Rondonópolis (de Lima Lemos et al. 2022) identified two factors associated with non‐vaccination, namely: having one or more siblings in the household (adjusted OR 3.18; IC 95% 1.75–5.76) and not receiving a visit from a community health agent in the last 30 days (adjusted OR 1.93; 95% IC 1.04–3.57).

In relation to racial inequalities, Boing et al. (2024) found that barriers to childhood vaccination were significantly greater among children of Black and Mixed race mothers compared to those of White mothers. This finding was based on an analysis of data from a national survey conducted in Brazil (2020–2022). For mothers of mixed race, the odds of facing difficulties in taking their children to vaccination rooms were 1.54 times higher (95% CI: 1.13–2.10) than for White mothers, after adjustment. Among Black mothers, this value was 1.54 times (95% CI: 1.10–2.16). Furthermore, even when reaching the health unit, children of Black and Mixed race mothers had 1.96 higher odds (95% CI: 1.56–2.45) and 1.92 higher odds (95% CI: 1.66–2.23), respectively, of not being vaccinated compared to children of White mothers (Boing et al. 2024).

In the present study, factors associated with IVS were found with important and statistically significant PR estimates for the variables: age (under 2 years), not receiving the vaccine in the same location and difficulties caused by the COVID‐19 pandemic in relation to vaccination.

Children under 2 years of age have a prevalence of IVS 2.55 times higher than older children. Among the possible explanatory hypotheses, it stands out that children who were under 2 years old at the time they were selected for the study, between February and March 2022, may have been more affected by changes in the functioning of BHU during the COVID‐19 pandemic, as this period coincided with the need to receive vaccines from the vaccination schedule under evaluation. However, the fact that the association remains after being adjusted for other variables, including the difficulties faced during the pandemic, indicates the existence of multiple explanatory factors, which are beyond the scope of this work.

A study carried out before the COVID‐19 pandemic, on factors associated with IVS in children up to 36 months of age in the city of Recife, in the State of Pernambuco, Brazil, identified that the older the age group, the greater the proportion of children with IVS. The authors indicate that the findings are probably justified because younger children have more opportunities to be vaccinated, as they attend health units more frequently, mainly due to the greater number of routine consultations, which, in turn, increases the opportunities to carry out vaccination (De Araújo Veras et al. 2020).

Reports of difficulties in vaccinating children during the COVID‐19 pandemic also showed a strong association with IVS (PR: 2.71, IC 95%: 1.51–4.86). The pandemic caused many different types of disruption to society, including the interruption of many health services (Nelson 2020). According to the World Health Organization (WHO), in 2020, with the restriction on immunization services, many parents/guardians stopped taking their children to be vaccinated, resulting in a reduction in VC (Organização Panamericana de Saúde—OPAS 2021).

According to Silveira et al. (2021), during the months of March to April 2020, the COVID‐19 pandemic caused a 20% decrease in the application of vaccines aimed at children in Brazil. This decline occurred at a time when the country was strongly advised to adopt social distancing measures. In this context, a recent literature review indicated that although the pandemic had a major impact on VC in Latin America, there was already a decline in VC before the pandemic, suggesting a complex and multifactorial causality (Castrejon et al. 2022). Therefore, while the pandemic has exacerbated the problem, Brazil's low vaccine coverage also reflects systemic gaps that began before 2020 (de Cantuária Tauil et al. 2024; Domingues et al. 2020).

Furthermore, there has been a lack of rigor in monitoring the vaccination schedule, especially, but not only, during the COVID‐19 pandemic. The lack of concern in updating the vaccination status may also have been influenced by the control of infectious diseases achieved with the PNI in 50 years of history, such as polio, measles and smallpox. Parents/guardians who have benefited from vaccines and who, therefore, have not lived with these diseases, may often not clearly understand the importance of immunization (Cruz 2017; Organização Panamericana de Saúde—OPAS 2021).

Regarding PHC attributes, although no statistically significant associations were identified between users' perception expressed through high or low scores and the IVS, possibly due to the homogeneity of how the population perceives the FHS teams evaluated, studies carried out with a greater number of teams and/or in other areas may find different results (Gomes et al. 2021; Starfield 2002).

The finding that not receiving the vaccine in the same location is associated with IVS reveals that the bond established with the vaccine room team, possibly established through longitudinal and accessible care, may be fundamental to achieving the VC recommended by the PNI. These results may also indicate that the evaluated population perceives that the service offered by the vaccination rooms is unrelated to the services offered by the FHS teams, which highlights a possible fragility of PHC in the region, given the importance of timely vaccination, during a routine consultation, for example, immediately referring to the vaccination room when a vaccination delay is identified (França et al. 2025; De Araújo Veras et al. 2020; Gomes et al. 2021).

In the PCATool analysis, the results demonstrate that affiliation, an attribute that assesses the bond with the family health team (Brasil, Ministério da Saúde 2020; Starfield 2002) presented an overall satisfaction score of 62.3%, for the first contact access‐accessibility attribute, which evaluates the health service in the agility of scheduling an appointment, the time spent for care, telephone assistance/guidance (Brasil, Ministério da Saúde 2020), the results were unsatisfactory, obtaining a low score in 90.7% of those interviewed.

These data indicate a weakness in the structure of the services, which may be related to the lack or low qualification of human resources, the lack of equipment, the difficulties in scheduling appointments, the waiting time of more than 30 min, the inadequate physical area for the service, generating physical barriers to accessibility (Araujo et al. 2018).

The data obtained with PCATool allowed capturing the user's self‐perception and even though it is a subjective assessment that varies according to individual understanding and experiences (Gomes et al. 2021), allowed measuring the presence and extent of attributes in the territory, which allows teams to remodel health practices.

Another important fact concerns subsequent doses of vaccines with more than one dose. In this study, vaccines with multi‐dose schedules showed a drop in vaccination percentages in subsequent doses, similar to what was found in other studies (Yokokura et al. 2013; de Miranda et al. 1995; Da Silva et al. 1999).

In vaccines for diseases that require more than one dose, there is a greater delay in vaccination in subsequent doses, which requires strategies to improve adherence, considering that the absence of administration of the last dose may not establish adequate immunity, leaving the individual prone, even if partially, to vaccine‐preventable diseases (Yokokura et al. 2013).

In this aspect, it is interesting to highlight that in the second half of 2019 there was a shortage of the pentavalent vaccine nationally (Domingues et al. 2023), coincidentally in this same period there was a drop in the number of doses of this vaccine administered in the country. This fact may justify the divergences found in this study regarding the doses of vaccines administered late at 2, 4, and 6 months, since they should have been administered simultaneously.

It is worth highlighting the relevance of factors associated with the current situation of low VC, including those reinforced and highlighted in the literature, such as difficulties in accessing health services (Duarte et al. 2018; de Barros et al. 2016), social vulnerability (Waldman 2008), anti‐vaccine movements (Cruz 2017; França et al. 2025), and the shortage of immunobiologicals (Domingues et al. 2023).

The main reasons for vaccine delay reported by those responsible in the present study were: the child being sick, forgetfulness, the COVID‐19 pandemic, difficulty in access, lack of vaccines and fear of adverse effects. Therefore, implementing strategies to reach a greater number of children vaccinated in a timely manner and reduce access barriers and lack of information are necessary. Health education strategies, active search, greater a community health agent involvement in home visits and checking the vaccination status of children under 2 years of age need to be strengthened, as indicated by other studies (Cruz 2017; França et al. 2025).

In this study, the lack of association between BHU that do or do not have a fixed vaccination room and IVS may have been influenced by the introduction of a mobile vaccination in one of the BHU in 2019. This fact may have facilitated access to vaccination, even with limitations regarding the availability of the service, as it is offered every 15 days. These and new immunization strategies must be formulated and established, such as extramural actions, for example, in schools and parks, so as not to leave the population unassisted, enabling access barriers to be overcome (Ministério da Saúde Secretaria de Vigilância em Saúde Departamento de Vigilância das Doenças Transmissíveis B 2014; Possas et al. 2020).

It is necessary to advance strategies that promote longitudinality and expanded access to immunization services, ensuring that these services are integrated with the assistance provided within the scope of PHC. It is understood that this will enable a broad awareness among those responsible for vaccinating children at the recommended age, prioritizing the correct administration of doses and, thus, reducing delays in vaccination. In this way, it will be possible, in addition to promoting disease control, to overcome barriers, including those imposed by the COVID‐19 pandemic, and potentially increase VC, as well as reduce and keep vaccine‐preventable diseases under control.

The main limitation of the study is the fact that it was not possible to reach the number of children predicted by the sample calculation, mainly due to the difficulty in locating those responsible and changes of address, which may have affected the precision of the study.

The main strengths of this study arise from the fact that it uses an easy‐to‐execute methodology to estimate the real VC, allowing replication in different locations. Furthermore, important factors associated with the incompleteness of the vaccination schedule were identified, reflecting the local reality and enabling actions to mitigate or eliminate them, which will potentially promote improvements in VC in the evaluated territories.

## 5 Conclusion

In the VC analysis for valid doses, the results were satisfactory, however, for the correct dose, no vaccine achieved the expected VC, indicating an important non‐compliance with the basic vaccination schedule, considering that, for successful immunization, it is necessary not only maintain the completeness of the vaccination schedule, but also strive to carry out the correct vaccination schedule.

The local factors associated with IVS were: age under 2 years, difficulties arising from the COVID‐19 pandemic and lack of continuity of vaccination in the same unit. The identification of these factors points to the need for local FHS teams to dedicate special attention, but not only, to children under 2 years of age. Furthermore, it is important that they offer a longitudinal vaccination service, accessible and integrated with the FHS, as well as making efforts to help the enrolled population overcome the difficulties imposed by the COVID‐19 pandemic. The importance of providing information about vaccination to parents/guardians and the general population is highlighted, in addition to expanding strategies to address access barriers.

## Author Contributions

I.R.M.N.V. and H.M.P. designed the study, constructed the database, analyzed the data and drafted the manuscript, and critically revised the manuscript. I.R.M.N.V. collected the data. All authors read and approved the final version of the manuscript.

## Funding

This study was supported with grants from the IATS‐CARE: Institute for Health Assessment and Translation for Chronic and Neglected Diseases of High Relevance ‐ CNPq/Brazil (408659/2024‐6). The article processing charge (APC) was funded by CAPES (Coordination for the Improvement of Higher Education Personnel) under the ROR identifier 00x0ma614.

## Ethics Statement

The research was approved by the Research Ethics Committee of the Faculty of Medicine of the University of Brasília—UnB. Opinion n° 5.508.477, CAAE n° 59336522.6.0000.5558. Only children whose guardians agreed to participate and signed the informed consent form were included in the study.

## Conflicts of Interest

The authors declare no conflicts of interest.

## Supporting information




**Supporting Figure 1**: phn70066‐sup‐0001‐figureS1.pdf

**Supplementary Box 1**: Vaccines from the basic vaccination schedule for children aged six months to under five years old, Ministry of Health, Brazil, 2020.

## Data Availability

The data that support the findings of this study are available on request from the corresponding author. The data are not publicly available due to privacy or ethical restrictions.
