# Supplementary material for: Vaccination Coverage and Factors Associated With Incomplete Vaccination Schedules in Children Under 5 in a Peripheral Area of the Federal District of Brazil
Source: Public Health Nurs. 2026 Jan 29;43(3):543–53. doi: 10.1111/phn.70066 (PMC13108635; doi:10.1111/phn.70066)
Supplement: Supplementary file 1 — Supporting Figure 1: phn70066‐sup‐0001‐figureS1.pdf [file PHN-43-543-s001.pdf]

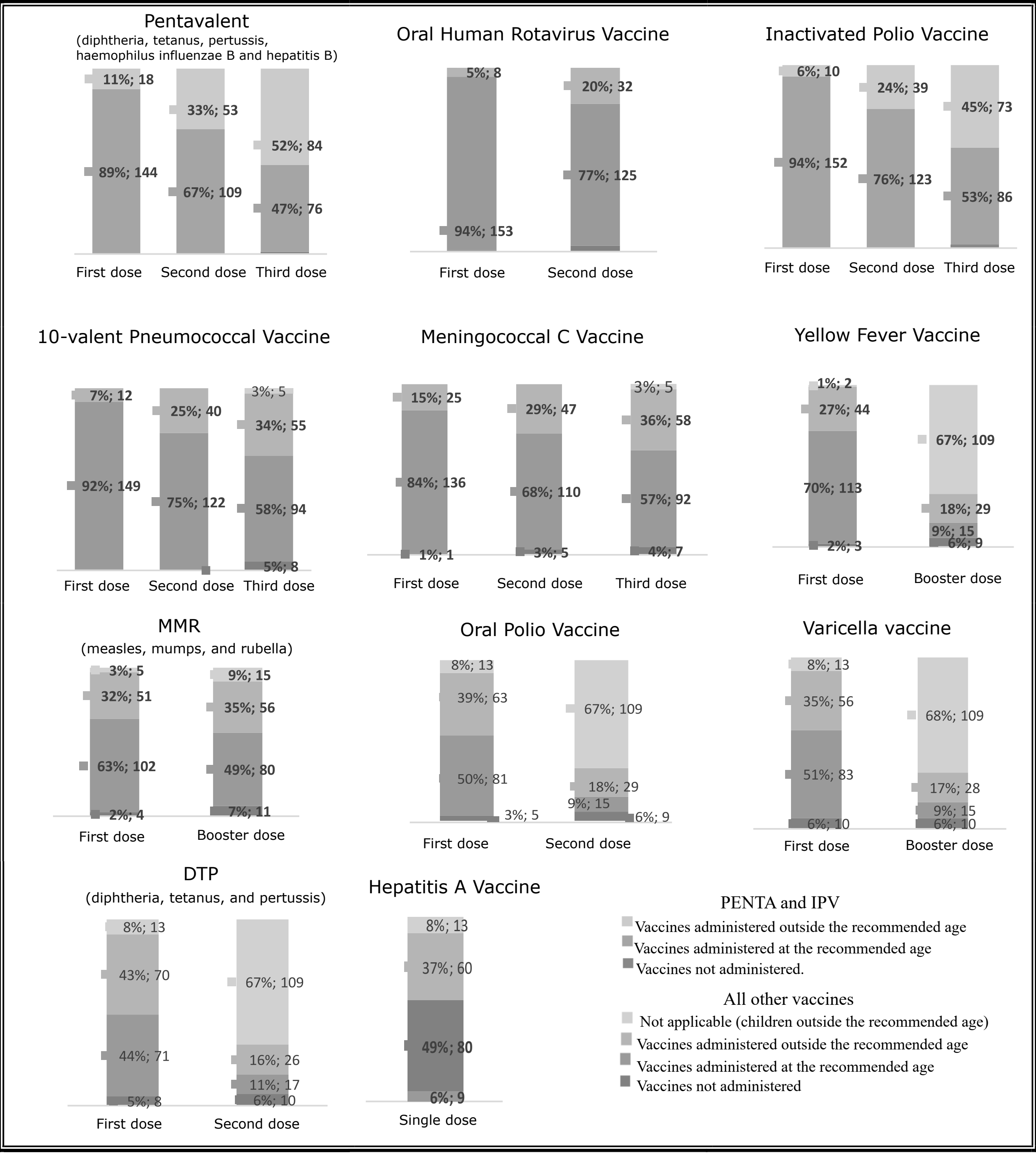
Pentavalent
(diphtheria, tetanus, pertussis, haemophilus influenzae B and hepatitis B)
11%; 18
89%; 144
33%; 53
67%; 109
52%; 84
47%; 76
First dose
Second dose
Third dose
Oral Human Rotavirus Vaccine
5%; 8
94%; 153
20%; 32
77%; 125
First dose
Second dose
Inactivated Polio Vaccine
6%; 10
94%; 152
24%; 39
76%; 123
45%; 73
53%; 86
First dose
Second dose
Third dose
10-valent Pneumococcal Vaccine
7%; 12
92%; 149
25%; 40
75%; 122
3%; 5
34%; 55
58%; 94
5%; 8
First dose
Second dose
Third dose
Meningococcal C Vaccine
15%; 25
84%; 136
1%; 1
29%; 47
68%; 110
3%; 5
3%; 5
36%; 58
57%; 92
4%; 7
First dose
Second dose
Third dose
Yellow Fever Vaccine
1%; 2
27%; 44
70%; 113
2%; 3
67%; 109
18%; 29
9%; 15
6%; 9
First dose
Booster dose
MMR
(measles, mumps, and rubella)
3%; 5
32%; 51
63%; 102
2%; 4
9%; 15
35%; 56
49%; 80
7%; 11
First dose
Booster dose
Oral Polio Vaccine
8%; 13
39%; 63
50%; 81
3%; 5
67%; 109
18%; 29
9%; 15
6%; 9
First dose
Second dose
Varicella vaccine
8%; 13
35%; 56
51%; 83
6%; 10
68%; 109
17%; 28
9%; 15
6%; 10
First dose
Booster dose
DTP
(diphtheria, tetanus, and pertussis)
8%; 13
43%; 70
44%; 71
5%; 8
67%; 109
16%; 26
11%; 17
6%; 10
First dose
Second dose
Hepatitis A Vaccine
8%; 13
37%; 60
49%; 80
6%; 9
Single dose
PENTA and IPV
Vaccines administered outside the recommended age
Vaccines administered at the recommended age
Vaccines not administered.
All other vaccines
Not applicable (children outside the recommended age)
Vaccines administered outside the recommended age
Vaccines administered at the recommended age
Vaccines not administered
