# Supplementary material for: Vaccination Coverage and Factors Associated With Incomplete Vaccination Schedules in Children Under 5 in a Peripheral Area of the Federal District of Brazil
Source: Public Health Nurs. 2026 Jan 29;43(3):543–53. doi: 10.1111/phn.70066 (PMC13108635; doi:10.1111/phn.70066)
Supplement: Supplementary file 2 — Supplementary Box 1: Vaccines from the basic vaccination schedule for children aged six months to under five years old, Ministry of Health, Brazil, 2020. [file PHN-43-543-s002.docx]

**Supplementary Box 1** - Vaccines from the basic vaccination schedule for children aged six months to under five years old, Ministry of Health, Brazil, 2020.

| **Vaccines** | **Target age**  **(months/year)** | **No. of doses of the vaccination schedule** | **Dose for calculating vaccination coverage** | **Vaccination coverage target** |
| --- | --- | --- | --- | --- |
| Pentavalent | 2, 4 and 6 months old | 3 | 3^rd^ DOSE | 95% |
| IPV | 2, 4 and 6 months old | 3 | 3^rd^ DOSE | 95% |
| ORV | 2 and 4 months old | 2 | 2^nd^ DOSE | 90% |
| PCV10 | 2, 4 and 12 months old | 3 | 2^nd^ DOSE | 95% |
| MenC | 3, 5 and 12 months old | 3 | 2^nd^ DOSE | 95% |
| Yellow fever vaccine | 9 months and 4 years old | 2 | 1^st^ DOSE | 95% |
| MMR | 12 and 15 months old | 2 | 1^st^ DOSE | 95% |
| Varicella vaccine | 15 months and 4 years old | 2 | 1^st^ DOSE | 95% |
| OPV | 15 months and 4 years old | 2 doses as booster | BOOSTER 1 | 95% |
| DTP | 15 months and 4 years old | 2 doses as booster | BOOSTER 1 | 95% |
| Hepatitis A vaccine | 15 months old | 1 | SINGLE DOSE | 95% |

Source: Ministry of Health, 2020. Pentavalent: Diphtheria, tetanus, pertussis, haemophilus influenzae B and hepatitis B vaccine; IPV: Inactivated polio vaccine; ORV: Oral human rotavirus vaccine; PCV10: 10-valent pneumococcal conjugate vaccine; MenC: Meningococcal C conjugate vaccine; MMR: Measles, mumps, and rubella vaccine; OPV: Oral polio vaccine; DTP: Diphtheria, tetanus, and pertussis vaccine.
